# Influence of Wounding and Temperature on Resistance of Maize Landraces From Mexico to Aflatoxin Contamination 

**DOI:** 10.3389/fpls.2020.572264

**Published:** 2020-09-24

**Authors:** Alejandro Ortega-Beltran, Peter J. Cotty

**Affiliations:** ^1^School of Plant Sciences, The University of Arizona, Tucson, AZ, United States; ^2^Agricultural Research Service, United States Department of Agriculture, Tucson, AZ, United States; ^3^School of Food Science and Engineering, Ocean University of China, Qingdao, China

**Keywords:** aflatoxin resistance, maize landrace, breeding for resistance, resistance components, post harvest resistance

## Abstract

Maize is a staple for billions across the globe. However, in tropical and sub-tropical regions, maize is frequently contaminated with aflatoxins by *Aspergillus* section Flavi fungi. There is an ongoing search for sources of aflatoxin resistance in maize to reduce continuous exposures of human populations to those dangerous mycotoxins. Large variability in susceptibility to aflatoxin contamination exists within maize germplasm. In Mexico, several maize landrace (MLR) accessions possess superior resistance to both *Aspergillus* infection and aflatoxin contamination but their mechanisms of resistance have not been reported. Influences of kernel integrity on resistance of four resistant and four susceptible MLR accessions were evaluated in laboratory assays. Wounds significantly (*P* < 0.05) increased susceptibility to aflatoxin contamination even when kernel viability was unaffected. Treatments supporting greater *A. flavus* reproduction did not (*P* > 0.05) proportionally support higher aflatoxin accumulation suggesting differential influences by some resistance factors between sporulation and aflatoxin biosynthesis. Physical barriers (i.e., wax and cuticle) prevented both aflatoxin accumulation and *A. flavus* sporulation in a highly resistant MLR accession. In addition, influence of temperature on aflatoxin contamination was evaluated in both viable and non-viable kernels of a resistant and a susceptible MLR accession, and a commercial hybrid. Both temperature and living embryo status influenced (*P* < 0.05) resistance to both aflatoxin accumulation and *A. flavus* sporulation. Lower sporulation on MLR accessions suggests their utilization would result in reduced speed of propagation and associated epidemic increases in disease both in the field and throughout storage. Results from the current study should encourage researchers across the globe to exploit the large potential that MLRs offer to breed for aflatoxin resistant maize. Furthermore, the studies provide support to the importance of resistance based on the living host and maintaining living status to reducing episodes of post-harvest contamination.

## Introduction

Aflatoxins are highly toxic and carcinogenic mycotoxins produced by *Aspergillus flavus* and closely-related fungi (Cotty et al., 1994). These potent toxins are detrimental to both human and animal health and one of them, aflatoxin B_1_, is classified as a Group 1 carcinogen by the International Agency for Research on Cancer (IARC) (JECFA, 2018). Concentrations of aflatoxins in foods and feeds are strictly monitored and regulated in most developed nations (Shephard, 2008; Wu, 2015; Udomkun et al., 2017).

Maize, the critical staple of billions, is frequently contaminated with aflatoxins. Therefore, populations relying on maize are at risk of chronic exposure (Plasencia, 2004; Bandyopadhyay et al., 2007; JECFA, 2018; Logrieco et al., 2018). Aflatoxin exposure is most severe in emerging and developing nations where legislation is poorly enforced or nonexistent (Resnik et al., 1995; Bandyopadhyay et al., 2016; Seetha et al., 2017; Logrieco et al., 2018). Because aflatoxin contamination of maize has little impact on yield, contamination may not attract farmer attention in the absence of strictly enforced and extensive awareness training (Bandyopadhyay et al., 2016; Udomkun et al., 2017).

Several management tools may be effective in limiting crop aflatoxin content (Bandyopadhyay et al., 2016; Seetha et al., 2017). It is preferred to utilize pre-harvest technologies, such as use of atoxigenic strains of *A. flavus* as biocontrol agents, to prevent initial aflatoxin formation. Another pre-harvest tool is to use maize cultivars resistant to both Aspergillus ear rot and aflatoxin accumulation (Warburton et al., 2009). However, high-yielding and aflatoxin resistant commercial maize hybrids are not available after over 40 years of research (Chen et al., 2010; Fountain et al., 2014; Warburton et al., 2015; Williams et al., 2015; Meseka et al., 2018; Suwarno et al., 2019). Development and large-scale use of maize cultivars with superior pre-harvest resistance to both fungal reproduction and aflatoxin accumulation may allow a low-cost and easily dispersed aflatoxin management alternative to complement aflatoxin biocontrol technologies.

Resistance to both biotic and abiotic factors is relatively common among landraces (Dwivedi et al., 2016). Although potentially useful variability exists in wild maize relatives and maize landraces (MLRs), that diversity remains largely untapped (Caldu-Primo et al., 2017; Romero Navarro et al., 2017), including for aflatoxin resistance (Warburton et al., 2017). Sources of resistance to both Aspergillus ear rot and aflatoxin accumulation are more likely to exist in maize adapted to both tropical and subtropical regions because of exposure to greater disease pressure (Menkir et al., 2006). MLR accessions from warm regions of Mexico have superior resistance to both kernel rot and aflatoxin accumulation (Ortega-Beltran et al., 2014). Identified resistant MLR accessions have potential to contribute to development of commercially acceptable, aflatoxin-resistant maize hybrids and/or open pollinated varieties. In order to achieve that, however, challenges to introgress MLR beneficial traits into advanced maize germplasm need to be resolved (Warburton et al., 2017).

Selection of MLRs over hundreds of generations by indigenous farmers in Mexico may have contributed to resistance to ear rot and, as a consequence, resistance to aflatoxin accumulation. Although aflatoxin resistant MLR accessions are known (Ortega-Beltran et al., 2014), the resistance mechanisms by which MLRs support lower aflatoxin content and reduced fungal growth remain unexplored. Kernel integrity, and wax and cutin content influence resistance of maize inbred lines to aflatoxin accumulation (Chen et al., 1998; Gembeh et al., 2001; Chen et al., 2004; Windham and Williams, 2007; Warburton et al., 2009). Temperature also influences crop susceptibility (Cotty and Jaime-Garcia, 2007; Medina et al., 2017) and a laboratory kernel screening assay (KSA) conducted at 31°C is useful for assessing maize susceptibilities to both fungal infection and aflatoxin contamination (Brown et al., 1993; Ortega-Beltran et al., 2014). However, in aflatoxin-prone areas, field temperatures before and during harvest, and throughout storage, may be substantially higher (Aidoo, 1993; Bruns, 2003; Hell et al., 2008; Fountain et al., 2014). It is unknown if maize characterized as aflatoxin resistant in KSA at 31°C will become susceptible at higher temperatures. In addition, kernels with damaged or dead embryos tend to accumulate greater aflatoxin content (Brown et al., 1993). Although the KSA has been broadly employed, the temperature dependence of embryo influences on aflatoxin accumulation remains uninvestigated.

In the present study, influences of physical barriers and kernel viability on resistance to both fungal reproduction and aflatoxin accumulation were investigated in accessions of MLRs. In addition, contributions of the living embryo to MLR resistance were quantified and the hypothesis that resistance in mature MLRs varies with temperature was tested. During the course of these investigations it was found that physical barriers are significant determinants of kernel vulnerability and MLR susceptibilities to both fungal reproduction and aflatoxin accumulation are influenced by temperature and maize kernel viability.

## Materials and Methods

### Maize Germplasm

Eight MLR accessions previously characterized as resistant or susceptible to aflatoxin contamination by *A. flavus* (Ortega-Beltran et al., 2014) were used in the current study. Three Tabloncillo (2006-04, 2006-23, and 2007-07) and one Vandeño (2007-06) accessions were classified as resistant (R-MLR). Two Tabloncillo (2006-04 and 2006-11), one Chapalote (2007-02), and one Reventador (2007-11) accessions were classified as susceptible (S-MLR). Shelled grains of these MLR accessions were imported into the US under an APHIS *Permit to Import Plant or Plant Products* and maintained at the USDA-ARS Aflatoxin Reduction in Crops Laboratory in the School of Plant Sciences, University of Arizona, Tucson. Samples were dried in a forced-air oven at 60°C for 48 h, stored in sealed bags and kept under refrigeration (4°C) until use. Grain of Pioneer maize hybrid P33B50 (from here on referred to as P33B50) supplied by Pioneer Hi-Bred International Inc. (Jhonston, IA) was used as positive control in two experiments.

### Fungal Isolates and Culture Conditions

*A. flavus* isolate AF13, recovered from agricultural soil in Arizona (Cotty, 1989), was used in three of the four experiments. AF13 consistently produces high aflatoxin B_1_ concentrations in different crops (Cotty, 1989; Brown et al., 1991; Brown et al., 1993; Mehl and Cotty, 2010) including the examined MLR accessions (Ortega-Beltran et al., 2014). For the temperature/living embryo status experiment, *A. flavus* isolate SSS06 MN-F, which belongs to vegetative compatibility group (VCG) SON003 from Sonora, Mexico, was used. Members of SON003 also produce large aflatoxin B_1_ concentrations and are common in agroecosystems where MLRs used in the current study are cultivated (Ortega-Beltran and Cotty, 2018; Ortega-Beltran et al., 2020). Inoculum suspensions were prepared by collecting spores from 5-day-old cultures (31°C) grown on 5-2 agar [5% V-8 juice (Campbell Soup Company, Camden, NJ), 2% Bacto-agar (Difco Laboratories Inc., Detroit, MI), pH 5.2] with a cotton swab into sterile distilled deionized water. Suspensions were quantified by turbidity using an Orbeco-Helling digital direct reading turbidimeter (Orbeco Analytical Systems Inc., Farmingdale, NY) and a Nephelometric Turbidity Unit (NTU) *versus* Colony Forming Unit (CFU) standard curve (*y* = 49,937*x; x* = NTU, *y* = spores ml^-1^) (Probst et al., 2010). Suspensions were diluted to a final concentration of 2 × 10^6^ spores ml^-1^.

### Preliminary Experiment

Mature kernels of each MLR accession and P33B50 were subjected to screening for post-harvest resistance to aflatoxin contamination and *A. flavus* reproduction using three kernel treatments: intact, incision in the embryonic region, and ground. Before treatment, damaged and undersized kernels were removed. Sorted kernels were surface-disinfected by immersion in hot water (80°C, 40 s). Then, kernels were blotted free of surface moisture in a biological safety cabinet for 15 min before treatment and transference to sterile 250-ml Erlenmeyer flasks. A sterile No. 12 Single Edge Industrial Razor Blade (VWR International, Westlake, LA) was used to make an incision in the center of the embryonic region with direction from the tip cap to the crown (approx. 1 mm deep, 2 mm long). For the ground treatment, embryo and kernel integrity were disrupted by grinding kernels in a laboratory analytical mill (Waring Commercial, Torrington, CT) for 15 s in a 110-ml stainless steel blending jar (MC-2).

Maize water content was measured with an HB43 Halogen Moisture Analyzer (Mettler Toledo, Columbus, OH). AF13 spore suspensions were combined with appropriate sterile water volumes to increase maize moisture content to 25%. Treatments were inoculated with approximately 350,000 spores g^-1^ of maize, covered with stoppers that allow gas exchange (BugStopper, Whatman, Piscataway, NJ) and incubated in a randomized block design (5 days, 31°C). Treatments inoculated with sterile water served as negative controls. Five kernels from each maize type (MLR accession and hybrid either ground, cut, or intact) were incubated (5 days, 31°C) on modified rose Bengal agar (MRBA) (Cotty, 1994) to assess kernel viability and absence of microorganisms. Two independent tests were conducted, each with four replicates. A replicate consisted of one flask containing 10 g of maize material.

### Influence of Incision Type and Kernel Integrity on Resistance of MLR 2006-23

To determine influences of damage to the embryo on resistance of R-MLR 2006-23, kernels were subjected to six independent treatments intended to disrupt the embryonic region. These included 1) the incision treatment described above; 2) bisecting kernels through the middle of the embryo with a razor blade; 3) grinding kernels as above; and 4) to 6) needle wounds to portions of the embryonic region. Kernel sorting, sterilization, grinding, and razor blade use were done as above. Needle wounds were made with a Precision Glide Needle IM 1½ 22GTW (BD, Franklin Lakes, NJ) to a depth of approx. 1 mm (Brown et al., 1993). Needle wounds in the embryonic region were located in the coleoptile (treatment 4), the scutellar node (treatment 5), and the coleorhiza (lower embryo, treatment 6). Preparation of AF13 spore suspensions, inoculation, and incubation were done as above. Five kernels from each treatment, except for the grinding treatment, were incubated on MRBA as above. Two independent tests consisting of five replicates each were conducted. A replicate consisted of a flask containing 10 g of maize material. Intact kernels of R-MLR 2006-23 served as controls.

### Influence of Wax and Cutin on Resistance of MLR 2006-23

Influences of chemical removal of kernel wax and cutin on maize kernel resistance to aflatoxin accumulation and *A. flavus* reproduction were quantified with R-MLR 2006-23. Intact kernels, ground, and ground and autoclaved kernels served as controls. Kernel sorting, surface-sterilization, and grinding were conducted as above. For the autoclaved treatment, ground kernels were autoclaved for 12 min at 121°C. Surface wax was removed by immersing kernels in three different washes of chloroform, 30 s each (Guo et al., 1995). The last treatment consisted of removal of wax as above followed by removal of cutin from the pericarp by immersing kernels into a 0.1M KOH solution for 30 min (Guo et al., 1995). Five kernels from each treatment, except for ground and ground-autoclaved kernels, were evaluated for viability and sterility on MRBA as above. Preparation of AF13 spore suspensions, inoculation, and incubation were conducted as above. Two independent tests consisting of five replicates each were performed. A replicate consisted of a flask containing 10 g of maize material.

### Influence of Temperature and Living Status on Resistance

Undamaged, mature kernels of S-MLR 2006-11, R-MLR 2006-23, and P33B50 were subjected to screening for support of both aflatoxin accumulation and *A. flavus* reproduction at three temperatures (25, 31, 35°C). Kernel sorting, surface-sterilization, and autoclaving were conducted as above. Five kernels from each treatment were evaluated for viability and sterility on MRBA as above. Preparation of SSS06 MN-F spore suspensions, inoculation, and incubation were conducted as previously. Two independent tests consisting of three replicates each were conducted. A replicate consisted of a flask containing 10 g of maize material.

### Aflatoxin Quantification

In all experiments, *A. flavus* growth was stopped after 5 days by adding 50 ml 70% methanol. Flasks were then swirled by hand (1 min) to dislodge the spores, and 1 ml of the mixture was removed for spore quantification. The remainder was ground as above. The blending jar was washed between replicates with 80% ethanol to avoid cross-contamination of aflatoxins. Homogenates were directly spotted (4 μl) in duplicate alongside aflatoxin standards (Supelco, Bellefonte, PA) on thin-layer chromatography (TLC) plates (Silica gel 60, EMD, Darmstadt, Germany) and developed with diethyl ether-methanol-water (96:3:1). Plates were visualized under UV light (365 nm), and presence or absence of aflatoxins was scored with the naked eye. Aflatoxins were quantified directly on TLC plates with a fluorescence scanning densitometer (CAMAG TLC Scanner 3, Muttenz, Switzerland) with software winCats 1.4.2, as previously described (Cotty and Cardwell, 1999). Samples from which aflatoxin B_1_ was not initially detected were combined with 100 ml water and extracted twice with 25 ml methylene chloride. Extracts were passed through a bed (25 g) of anhydrous sodium sulfate contained in fluted Whatman No. 4 filter paper, combined, and evaporated to dryness (Cotty and Cardwell, 1999). Residues were solubilized in an appropriate volume of methylene chloride for accurate densitometry and quantified as above. The limit of detection was 20 parts per billion (ppb) of aflatoxin B_1_.

### Variability in Support of *Aspergillus flavus* Reproduction

In order to gain insight into fungal reproduction by *A. flavus* isolates on different treatments in each test, production of spores was quantified. Spore suspensions (1 ml) washed from infected kernels were diluted 20-fold with 50% methanol, and turbidity was quantified in NTUs as described above.

### Statistical Analyses

In all experiments, results from two independent tests were similar allowing results to be combined. All statistical tests were performed using SAS 9.2 (SAS Institute, Cary, NC). Aflatoxin B_1_ concentrations in ppb and *A. flavus* reproduction in NTU g^-1^ were log-transformed to normalize variances; aflatoxin B_1_ values in the text and the tables, however, are expressed in parts per million (ppm). Experiments were performed with completely randomized designs, and the resulting data was subjected to Analysis of Variance (ANOVA). Means among treatments and within maize entries were separated using Fisher’s Least Significant Difference (LSD) test (α=0.05). In the preliminary experiment, a repeated measures approach was conducted to test for differences between ground kernels and kernels with incision treatments. Pearson’s correlation analysis using the CORR procedure was used to determine relationships between aflatoxin B_1_ accumulation and *A. flavus* reproduction. In the temperature experiment the data was subjected to a Factorial Analysis of Variance to compare the main effects of temperature (25, 31, and 35°C), maize living status (dead and alive), maize type (S-MLR 2006-11, R-MLR 2006-23, and P33B50), and the interaction effects within and among those variables on both aflatoxin accumulation and fungal reproduction (LSD test, α=0.05). Comparisons of both aflatoxin accumulation and fungal reproduction between viable and non-viable kernels at each temperature were performed with Student’s *t*-test (α=0.05). The aflatoxin B_1_ value of R-MLR 2006-23 living kernels at 25°C was below the limit of detection but was assigned to 20 ppb for the purpose of calculating the percent reduction in comparison with dead kernels at 25°C.

## Results

### Preliminary Experiment

All kernels were free of contaminants, as a result of the surface disinfection. In intact kernels, aflatoxin B_1_ produced by *A. flavus* AF13 was significantly (*P* < 0.0001) lower in the four R-MLRs than in S-MLRs and P33B50, as expected (**Table 1**). Significant differences among R-MLRs were also detected. Among the susceptible germplasm, S-MLR 2006-04 was the sole accession that significantly (*P* < 0.0001) differed from P33B50. R-MLR 2006-23 supported undetectable aflatoxin levels. Lower (*P* < 0.0001) sporulation occurred in all MLR accessions (at least 92% less) compared to P33B50 although differences were detected among resistant and susceptible MLR accessions (**Table 1**). A significant correlation (R = 0.7320, *P* = 0.0250) was detected between aflatoxin accumulation and fungal reproduction in intact MLR kernels. Overall, the intact R-MLR accessions averaged over 10-fold lower aflatoxin concentrations than the intact S-MLR accessions (2.0 ppm *vs* 26.5 ppm; *P* < 0.05, Student’s *t*-test).

**Table 1:** Aflatoxin B_1_ and spore production by *Aspergillus flavus* AF13 in kernels of resistant and susceptible maize landrace accessions, four of each, and commercial hybrid P33B50 that were either intact, with an incision, or ground.

Maize^t^	Race^u^	Class^v^	Aflatoxin B_1_ (ppm)^w^				Spore production (NTU g^-1^)^y^			
			Intact	Incision	Ground	Within accessions^x^	Intact	Incision	Ground	Within accessions^z^
2006-04	Tabloncillo	S	19 b	166 ab	95 ab	B, A, A*	2.4 bc	10.1 cd	44 d	C, B, A**
2006-11	Tabloncillo	S	29 ab	143 bc	50 b	B, A, A*	2.9 bc	7.1 de	46 cd	C, B, A**
2007-02	Chapalote	S	38 a	169 ab	46 b	B, A, B*	3.8 b	17.0 bc	55 c	C, B, A**
2007-11	Reventador	S	20 ab	150 b	40 b	C, A, B*	3.8 b	23.1 b	86 ab	C, B, A**
2006-10	Tabloncillo	R	2 cd	166 ab	150 a	B, A, A*	2.1 cd	17.9 b	83 ab	C, B, A**
2006-23	Tabloncillo	R	0 e	185 ab	51 b	C, A, B**	1.2 d	9.3 cd	82 b	C, B, A**
2007-06	Vandeño	R	5 cd	216 a	85 ab	B, A, A*	3.8 b	5.6 e	50 cd	B, B, A**
2007-07	Tabloncillo	R	1 d	107 c	55 b	B, A, A**	3.1 bc	19.2 b	99 a	C, B, A**
P33B50	–	C	54 a	211 a	43 b	B, A, B*	48.0 a	58.9 a	88 ab	C, B, A**

^t^Code used to identify accessions. Year collected followed by a serial number.

^u^Morphological characteristics were used to assign maize accessions to their corresponding maize landrace (MLR) (Sanchez et al., 2000).

^v^Classification based on susceptibility to aflatoxin accumulation in a previous study (Ortega-Beltran et al., 2014). R: resistant, S: susceptible, C: commercial hybrid.

^w^Values, in parts per million (ppm), correspond to aflatoxin B_1_ means of 10 replicates from two independent similar tests. In each treatment, values with different lower-case letters indicate significant differences for log transformed data of aflatoxin B_1_ ppm by Fisher’s LSD (α=0.05).

^x^Comparison among treatments in each maize entry. Different upper-case letters indicate significant differences for log transformed data of aflatoxin B_1_ ppm by Fisher’s LSD (α=0.05). A single asterisk indicates P < 0.05; triple asterisks indicate P < 0.0001. For example, aflatoxin contents in MLR 2006-04 among the three treatments (Intact, Incision, Ground) were compared and it was found that the intact treatment had significantly less (P < 0.05) aflatoxin than the two other treatments.

^y^Values are AF13 reproduction rates in NTU g^-1^ (NTUs: nephelometric turbidity units, 1 NTU = 49,937 spores). Values are means of eight replicates from two independent similar tests. In each treatment, values with different lower-case letters indicate significant differences for log transformed data of NTUs by Fisher’s LSD (α=0.05).

^z^Comparison of fungal reproduction among treatments in each maize entry. Different upper-case letters indicate significant differences for log transformed NTU by Fisher’s LSD (α=0.05). A single asterisk indicates P < 0.05; two asterisks indicate P < 0.0001.

Aflatoxin concentrations in kernels with an incision varied (*P* < 0.0001) among and within resistant and susceptible accessions (**Table 1**). Embryo viability was not affected by the incision, but susceptibilities to contamination increased in all MLRs. P33B50 was among the entries with highest aflatoxin concentrations. Significantly (*P* < 0.05) lower aflatoxin content occurred in R-MLR 2007-07 than in the rest of the entries, except S-MLR 2006-11. Fungal reproduction in each MLR accession was significantly (*P* < 0.0001) lower than in P33B50, although differences were detected among and within resistant and susceptible accessions (**Table 1**). There was no significant correlation between aflatoxin accumulation and fungal reproduction (R = 0.2734, *P* = 0.4766) in kernels with an incision. On average, incised R-MLR (168 ppm) and incised S-MLR (157 ppm) accessions did not differ significantly (*P* > 0.05, Student’s *t*-test).

Aflatoxin accumulation was greater (*P* = 0.0327, Fisher’s protected LSD) in ground kernels of one R-MLR than in three S-MLR accessions and P33B50 (**Table 1**). Significant differences (*P* < 0.0001) in fungal reproduction were detected among ground maize entries (**Table 1**). Fungal reproduction and aflatoxin accumulation were not correlated (R = -0.0926, *P* = 0.5911) in ground kernels.

Generally, lower aflatoxin content occurred in intact kernels (average = 19 ppm) compared to kernels with an incision (average = 168 ppm) and ground kernel treatments (average = 68 ppm) except for S-MLR 2007-02 and P33B50, in which aflatoxin content was similar (*P* < 0.05) in intact and ground kernels (**Table 1**). Five of the eight MLR accessions supported statistically similar aflatoxin levels in kernels with an incision and ground kernels (**Table 1**). R-MLR 2006-23 and S-MLR 2007-11 were the sole accessions in which all treatments were significantly (*P* < 0.001) different, with intact kernels supporting the lowest and kernels with an incision the highest aflatoxin content. Significant (*P* < 0.001) differences in susceptibility to fungal growth were detected within each maize accession (**Table 1**). The lowest fungal growth occurred in intact kernels, and the maximum occurred in ground kernels, except for R-MLR 2007-06 in which similar levels occurred in intact kernels and kernels with an incision. On average, kernels with an incision supported significantly (*P* < 0.0001) greater aflatoxin production than ground kernels (168 ppm *vs.* 68 ppm) and significantly (*P* < 0.0001) less fungal reproduction (75% less).

### Influence of Incision Type and Kernel Integrity in Resistance of MLR 2006-23

Accession R-MLR 2006-23 exhibited the greatest intact kernel resistance in the preliminary experiment (**Table 1**) and therefore, was selected for additional studies on effects of wounding on both aflatoxin accumulation and fungal reproduction. After surface sterilization, all kernels were free of contaminants. Embryo viability was eliminated in bisected and ground kernels. Aflatoxin content was significantly (*P* < 0.0001) lower in intact kernels, 2 ppm. Significant differences among the other treatments were not detected (**Table 2**). However, when excluding the intact kernels treatment from the Analysis of Variance, Fischer’s LSD revealed significant (*P* < 0.0001) differences among kernels with compromised tissue integrity (**Table 2**). Ground kernels supported the lowest aflatoxin content (64 ppm). Bisected kernels accumulated aflatoxin levels similar to those accumulated by kernels subjected to each pin-wounded treatment, but significantly (*P* < 0.0001) less than kernels with an incision (**Table 2**). Significantly (*P* < 0.0001) lower fungal reproduction occurred in intact kernels, as expected, than in other treatments (**Table 2**). The greatest fungal reproduction occurred in ground kernels, but this treatment yielded the least aflatoxin content (**Table 2**). No significant differences in fungal growth were detected among kernels with any of the four types of incision in the embryonic region.

**Table 2:** Aflatoxin B_1_ and spore production by *Aspergillus flavus* AF13 in kernels of R-MLR 2006-23 subjected to seven treatments.

Treatment	Aflatoxin B_1_ (ppm)^y^	Spore production (NTU g^-1^)^z^
Intact	2 b	1.1 d
Incision	185 a, *a*	9.3 c
Cut in half	114 a, *b*	37.1 b
Ground	64 a, *c*	87.2 a
Needle pin above embryo	140 a, *ab*	8.5 c
Needle pin center of embryo	115 a, *b*	8.8 c
Needle pin below embryo	140 a, *ab*	8.6 c

^y^Values, in parts per million (ppm), correspond to aflatoxin B_1_ means of 10 replicates from two independent similar tests. In each treatment, values with different lower-case letters indicate significant differences for log transformed data of aflatoxin B_1_ ppm by Fisher’s LSD (α=0.05). Lower-case letters in italics following the comma indicate significant differences among treatments excluding intact treatment.

^z^Values are AF13 reproduction rates in NTU g^-1^ (NTUs: nephelometric turbidity units, 1 NTU = 49,937 spores). Values are means of 10 replicates from two independent similar tests. Values with different lower-case letters indicate significant differences for log transformed data of NTUs by Fisher’s LSD (α=0.05).

### Influence of Wax and Cutin on Resistance of MLR 2006-23

Further evaluation of mechanisms of resistance in R-MLR 2006-23 yielded significant (*P* < 0.0001) differences in aflatoxin accumulation between intact kernels (no aflatoxin detected) and the other treatments (**Table 3**). Kernels from each of this experiment’s treatments were free of contaminants and embryo viability was affected only in ground and ground-autoclaved kernels. Aflatoxin content was higher in kernels with both wax and cuticle removed than in kernels with only wax removed. Higher aflatoxin content occurred in ground-autoclaved kernels than in ground kernels when comparing these treatments separately (*P* < 0.05, Student’s *t*-test, data not shown). Means separation of treatments was not affected by exclusion of the intact kernels’ treatment (data not shown). Fungal reproduction increased as physical barriers were removed (**Table 3**). Fungal reproduction was significantly (*P* < 0.0001) greater in both ground and ground-autoclaved kernels than in the rest of the treatments.

**Table 3:** Aflatoxin B_1_ and spore production by *Aspergillus flavus* AF13 in kernels of R-MLR 2006-23 subjected to five treatments.

Treatment	Aflatoxin B_1_ (ppm)^y^	Spore production (NTU g^-1^)^z^
Intact	0 c	1 d
Ground	39 ab	125 a
Ground autoclaved	65 a	131 a
Wax removed	23 b	7 c
Wax and cuticle removed	52 a	14 b

^y^Values, in parts per million (ppm), correspond to aflatoxin B_1_ means of 10 replicates from two independent similar tests. In each treatment, values with different lower-case letters indicate significant differences for log transformed data of aflatoxin B_1_ ppm by Fisher’s LSD (α=0.05).

^z^Values are AF13 reproduction rates in NTU g^-1^ (NTUs: nephelometric turbidity units, 1 NTU = 49,937 spores). Values are means of 10 replicates from two independent similar tests. Values with different lower-case letters indicate significant differences for log transformed data of NTUs in spores g^-1^ by Fisher’s LSD (α=0.05).

### Influence of Temperature and Living Status on Resistance

Both surface-sterilized living and autoclaved kernels were free of contaminants and only autoclaved kernels were non-viable. Significant differences (*P* < 0.05) in aflatoxin accumulation were detected among maize entries at each temperature, regardless of living status, except for living kernels at 25°C (*P* = 0.3557; **Table 4**). In living kernels at 31°C, susceptibilities to aflatoxin contamination among maize entries were maintained as in our previous study (Ortega-Beltran et al., 2014) and the preliminary experiment (**Table 1**). On the other hand, resistance of R-MLR 2006-23 was greatly affected at 35°C where it accumulated aflatoxin concentrations similar to S-MLR 2006-11 (**Table 4**). The two MLR accessions accumulated lower aflatoxin accumulation than P33B50 in living kernels at 35°C, but the opposite occurred in dead kernels (*P* = 0.0496). R-MLR 2006-23 allowed 97.8% more aflatoxins in living kernels at 35°C than at 31°C but 15.7% more aflatoxins in dead kernels at 31°C than at 35°C. S-MLR 2006-11 allowed similar aflatoxin concentrations at 31 and 35°C. Autoclaving kernels had a profound effect on aflatoxin accumulation. Each maize entry, at each temperature, had greater (*P* < 0.05) aflatoxin accumulation in dead kernels (**Table 4**). In R-MLR 2006-23, at 25 and 31°C, over a 99.5% aflatoxin accumulation differential occurred in dead kernels while a 70.8% differential occurred at 35°C. Aflatoxin accumulation in dead R-MLR 2006-23 kernels was consistently among the highest (*P* < 0.05), regardless of temperature. Aflatoxin accumulation differentials progressively reduced as temperature increased although this occurred to a greater extent in P33B50 kernels (**Table 4**).

**Table 4:** Comparison of aflatoxin B_1_ production by *Aspergillus flavus* SSS06 MN-F in living versus dead kernels of two native maize landrace (MLR) accessions and a commercial hybrid at three different temperatures.

Maize^u^	25°C^x^			31°C^x^			35°C^x^		
	Aflatoxin B_1_ (ppm)		Avg. diff. (%)^y^	Aflatoxin B_1_ (ppm)		Avg. diff. (%)^y^	Aflatoxin B_1_ (ppm)		Avg. diff. (%)^y^
	Living	Dead		Living	Dead		Living	Dead	
S-MLR 2006-11	0.4 a*	3.0 b	86.7	21.3 a**	121.2 a	82.4	20.6 b*	80.3 a	74.3
R-MLR 2006-23	0.0 a**	11.3 ab	99.9	0.5 b*	91.2 ab	99.5	23.0 b**	78.8 a	70.8
P33B50	0.6 a*	14.7 a	95.9	30.6 a*	73.9 b	45.1	35.8 a*	59.8 b	40.1
*P*-value^z^	0.3557	0.0347		<0.0001	0.0355		0.0066	0.0496	

^u^Code used to identify accessions used in the current study. Year collected followed by a serial number, and Pioneer hybrid P33B50.

^v^Maize accessions were classified as belonging to MLRs based on morphological characteristics (Sanchez et al., 2000).

^w^This classification of MLRs was based on previous studies in variation in susceptibility to aflatoxin accumulation when comparing to a standard hybrid control at 31°C (Ortega-Beltran et al., 2014).

^x^Values, in parts per million (ppm), correspond to aflatoxin B_1_ means of six replicates from two independent similar tests. Values with different lower-case letters indicate significant differences among maize entries in aflatoxin B_1_ accumulation by Fisher’s LSD (α=0.05) for log transformed data of aflatoxin B_1_ at each temperature for each maize living status. Differences between living and dead kernels for each maize at each temperature were tested for significance with Student’s t-test; significance (P < 0.05) is indicated by a single asterisk or two asterisks for P < 0.001.

^y^Reduction was calculated as the percentage of the proportion of aflatoxin B_1_ accumulation in living kernels in comparison to that of dead kernels.

^z^P-value of means separation in each temperature for the corresponding maize embryo living status with Fisher’s LSD (α=0.05).

Aflatoxin accumulation significantly differed when the tested variables (maize type, embryo status, temperature) were analyzed independently of each other. Aflatoxin accumulation was lowest in R-MLR 2006-23, followed by S-MLR 2006-11, and the highest occurred in P33B50 (*P* < 0.001, Mixed models). Aflatoxin content increased in living kernels with temperature (*P* < 0.0001, Mixed models). However, in dead kernels, maximum aflatoxin accumulation occurred at 31°C. Interaction among any two of the three tested variables and the combined three variables was significant (*P* < 0.05, Mixed models).

There were significant (*P* < 0.05) differences among maize entries in support of *A. flavus* reproduction at each temperature in both dead and living maize kernels (**Table 5**). At 25°C, *A. flavus* reproduction was greater (*P* < 0.0001, Student’s *t*-test) in dead than in living kernels of P33B50 while living and dead kernels of each of the two MLR accessions had similar fungal reproduction levels. On the other hand, at 31°C significantly higher (*P* < 0.0001, Student’s *t*-test) fungal reproduction occurred in dead kernels of the two MLR accessions but not in P33B50. Further, significantly (*P* < 0.0001, Student’s *t*-test) greater fungal reproduction occurred in dead kernels of each maize entry at 35°C. In the two MLR accessions, fungal reproduction differentials increased with temperature (**Table 4**). P33B50 supported the most reproduction by *A. flavus* at each temperature with the greatest difference on living kernels occurring at 35°C where *A. flavus* produced greater than 6-fold more spores on P33B50 than on either of the MLR accessions.

**Table 5:** Comparison of *Aspergillus flavus* SSS06 MN-F reproduction in living versus dead kernels of two native maize landrace (MLR) accessions and a commercial hybrid at three different temperatures.

Maize^u^	25°C^x^			31°C^x^			35°C^x^		
	Spores (NTU g^-1^)		Avg. diff. (%)^y^	Spores (NTU g^-1^)		Avg. diff. (%)^y^	Spores (NTU g^-1^)		Avg. diff. (%)^y^
	Living	Dead		Living	Dead		Living	Dead	
S-MLR 2006-11	2.5 ab	2.5 b	0.0	17.8 b**	50.8 a	64.9	7.0 b**	54.2 b	87.1
R-MLR 2006-23	2.1 b	2.5 b	16.0	4.3 c**	30.2 b	85.8	3.3 c**	26.1 c	87.4
P33B50	2.7 a**	11.4 a	76.3	43.4 a	44.3 a	2.0	43.7 a**	74.8 a	41.6
*P*-value^z^	0.1129	<0.0001		<0.0001	0.0003		<0.0001	<0.0001	

^u^Code used to identify accessions used in the current study. Year collected followed by a serial number, and Pioneer hybrid P33B50.

^v^Maize accessions were classified as belonging to MLRs based on morphological characteristics (Sanchez et al., 2000).

^w^This classification of MLRs was based on previous studies in variation in susceptibility to aflatoxin accumulation when comparing to a standard hybrid control at 31°C (Ortega-Beltran et al., 2014).

^x^Values are SSS06 MN-F reproduction rates in NTU g^-1^ (NTUs: nephelometric turbidity units, 1 NTU = 49,937 spores). Values are means of six replicates from two independent similar tests. Values with different lower-case letters indicate significant differences among maize entries A. flavus reproduction by Fisher’s LSD (α=0.05) for log transformed data of NTUs at each temperature for each maize living status. Differences between living and dead kernels for each maize at each temperature were tested for significance with Student’s t-test; significance (P < 0.05) is indicated by a single asterisk or two asterisks for P < 0.001.

^y^Reduction was calculated as the percentage of the proportion of A. flavus reproduction in living kernels in comparison to that in dead kernels.

^z^P-value of means separation in each temperature for the corresponding maize embryo living status with Fisher’s LSD (α=0.05).

Significant differences were detected in fungal reproduction when analyzing each of the tested variables independently of each other. Fungal reproduction was lowest in R-MLR 2006-23, at 25°C, and in living kernels (*P* < 0.0001, Mixed models). Interaction of any two of the three tested variables and the combined three variables was highly significant (*P* < 0.0001, Mixed models).

## Discussion

In Mexico, MLRs adapted to diverse environments are used by smallholder farmers because of good local performance, and social and cultural preferences (Hellin et al., 2014; Mastretta-Yanes et al., 2018; Johnson, 2020). Typically, landraces of any given crop perform relatively well when challenged with biotic and abiotic stresses (Dwivedi et al., 2016) and this contributes to MLR preference for cultivation. MLRs with superior resistance to both kernel rot and aflatoxin accumulation have previously been reported from two regions in Northwest Mexico (Ortega-Beltran et al., 2014). In the current study, influences of physical damage and environmental conditions on aflatoxin accumulation in MLR accessions previously classified as resistant or susceptible were examined to gain insight on characteristics leading to resistance. The overall results suggest that practices that maintain intact viable kernels until utilization will result in decreased aflatoxin contamination. Resistance to aflatoxin accumulation declined when kernels of MLR accessions either lost viability or were subjected to minor wounds and loss of physical barriers. Further, elevated temperature was found to increase susceptibility to aflatoxin contamination of an otherwise resistant MLR accession. However, resistant and susceptible MLR accessions accumulated less aflatoxins than a commercial hybrid at 35°C, the highest temperature tested.

The initial experiment, in the current work, demonstrated that MLRs characterized as resistant or susceptible to aflatoxin accumulation in intact viable kernels retained the relative susceptibility to contamination originally observed (Ortega-Beltran et al., 2014). However, a single small incision in the embryonic region of kernels reduced resistance in both susceptible (S-MLR) and resistant (R-MLR) accessions resulting in a 4- to 9-fold increase in aflatoxin accumulation in S-MLR accessions and a 43- to 100-fold increase in R-MLR accessions (**Table 1**). This suggests that the resistance mechanism overcome by the incision is present, but at different magnitudes, in both the S-MLR and R-MLR accessions. By pin-wounding the embryo region of kernels of marker-assisted-selection populations, Brown et al. (Brown et al., 1993) found higher *A. flavus* reproduction and greater aflatoxin accumulation compared to intact kernels. Guo et al. (Guo et al., 1995) also found that inflicting damage to kernel pericarp was associated with reduced resistance to both fungal reproduction and aflatoxin accumulation. The presented results indicate that kernel integrity is fundamental to both maintain resistance to aflatoxin contamination and to reduce susceptibilities to fungal reproduction in most MLRs (**Table 1**).

Among diverse maize genotypes, kernel architecture has different influences in resistance to fungal reproduction and aflatoxin accumulation. Mellon et al. (2005) reported that both aflatoxin production and fungal growth were reduced in ground maize. Intact kernel architecture in those genotypes favored both aflatoxin biosynthesis and fungal growth. In the current studies, ground MLR kernels supported on an average only 44% as much aflatoxin as kernels that only received an incision. However, fungal reproduction on ground MLR kernels was on an average 5-fold greater than on incised kernels (**Table 1**). Fungal reproduction on the ground and incised kernels of the improved hybrid supported similar levels of fungal reproduction. Causes of increased fungal growth in ground MLR kernels but not in those of improved maize remain unclear. Ground kernels had less aflatoxin than kernels with an incision despite increased access to carbohydrates but supported more fungal reproduction. In the evaluated MLRs, when relatively small lesions were inflicted, kernel architecture favored aflatoxin accumulation. If insect and/or mechanical damage occurs during or after harvest of the examined MLRs, it would be safer to store the grain as flour to prevent aflatoxin contamination. This, however, should be accompanied by good storage practices with maintenance of low water activity to reduce susceptibilities to fungal growth. On the other hand, maize preparation of many traditional dishes (e.g., artisanal tortillas, pozole, menudo) requires use of whole kernels (Nuss and Tanumihardjo, 2010; Ranum et al., 2014) and processing all of the maize produced by a household into flour is not a viable practice. A more acceptable manner for maintaining maize grain quality in households may be use of small-scale hermetic storage devices (Odjo et al., 2020).

R-MLR 2006-23 was selected to investigate influences of different types of damage on both aflatoxin accumulation and fungal reproduction. Damage to the kernel resulted in both increased *A. flavus* sporulation and increased aflatoxin production. However, complete disruption of kernel structure resulted in less aflatoxin production and more sporulation than relatively small lesions to the embryonic region (**Table 2**). Brown et al. (1993) similarly found that pin wounds in the embryonic region increased aflatoxin accumulation in a resistant maize population from Mississippi, United States. In the current study, aflatoxin accumulation in bisected kernels could be distinguished from accumulation in both ground and kernels with a razor blade incision. Indeed, the greater the disruption of kernel compartmentalization, the lower the aflatoxin accumulation with 64 ppm, 114 ppm, and 185 ppm in ground kernels, bisected kernels, and incised kernels, respectively, even though the incision treatment was the only one that did not eliminate kernel viability (**Table 2**). Keller et al. (1994) found aflatoxin biosynthesis compartmentalized in embryonic regions. Taken together with the current results, factors compartmentalized in whole kernels may be antagonistic to aflatoxin biosynthesis but not *A. flavus* growth and reproduction. Candidate factors may have been previously identified (Chen et al., 1998; Gembeh et al., 2001). Pin holes in several locations in the embryo region yielded similar results for both aflatoxin and fungal reproduction (**Table 2**). Thus, pin holes may serve primarily in providing access. The components dictating the contributions of distinct kernel tissues to aflatoxin resistance should be further investigated.

Physical barriers are important to resist both aflatoxin accumulation and fungal reproduction (**Table 3**). Lower aflatoxin levels were detected in living tissues than in nonliving tissues of ground kernels when comparing these two treatments separately (data not shown), but no differences in fungal reproduction were detected (**Table 3**). Similar observations have been previously made (Brown et al., 1993; Probst and Cotty, 2012). Aflatoxin accumulation and fungal reproduction increased as wax and cuticle were removed (**Table 3**). Both wax and cuticle are important contributors to resistance in other maize genotypes (Guo et al., 1995). Kernel wax from certain maize genotypes inhibit *A. flavus* growth (Guo et al., 1995; Russin et al., 1997; Gembeh et al., 2001) although the current study did not test inhibitory effects of MLR wax on *A. flavus* growth.

Lipoxygenases of soybean have been associated with increased resistance to *A. flavus* infection (Mellon and Cotty, 2002). Lipoxygenase activity is terminated by autoclaving. In addition, autoclaving seeds disrupts kernel integrity by exposing seeds to breaks, leaving kernels more susceptible to fungal infection and subsequent aflatoxin production (Mellon and Cotty, 2002). Further, wax and cuticle from kernel pericarp play important roles as defensive barriers against fungal infection and these two components may be altered during the autoclaving process (Guo et al., 1995; Russin et al., 1997; Gembeh et al., 2001). Whether the observed increases in susceptibility to aflatoxin contamination in MLRs was a result of heat treatment by autoclaving (which affected embryo viability) or due to disruption of both wax and cutin layers should be further investigated.

Several studies have screened maize entries for post-harvest resistance at a set temperature with mature kernel assays (Brown et al., 1993; Campbell and White, 1995; Guo et al., 1995; Tubajika and Damann, 2001; Menkir et al., 2006; Ortega-Beltran et al., 2014). In the current study, we examined susceptibility of maize kernels to aflatoxin accumulation at three different temperatures using a KSA. Resistance was found to be temperature-dependent for both susceptible and resistant MLR accessions and for the commercial hybrid (**Table 4**). On average, susceptibility increased with temperature from 3 ppm at 25°C to 26 ppm at 35°C. At each temperature for all three maize types examined, living kernels were more resistant to aflatoxin accumulation than dead kernels (**Table 4**). These results agree with previous suggestions that metabolic activities of the living maize embryo contribute substantially to postharvest resistance to aflatoxin contamination (Brown et al., 1993). Regardless of the level of resistance observed in each entry at each temperature, it is evident that living maize embryos of both MLR accessions and P33B50 are fundamental to protect against aflatoxin accumulation and fungal reproduction. At 31°C, living kernels of all entries accumulated similar aflatoxin concentrations as in previous studies at 31°C (Ortega-Beltran et al., 2014). Low aflatoxin in living kernels tested at 25°C was expected because this is below optimum temperature range for successful growth and aflatoxin production by *A. flavus* in living substrates (Cotty et al., 1994). On the other hand, it was unexpected that the relative resistance in R-MLR 2006-23 would be temperature-dependent. Differences in susceptibility at 31°C between S-MLR 2006-11 and R-MLR 2006-23 were lost at 35°C and those between R-MLR 2006-23 and P33B50 were greatly diminished. However, at 35°C, aflatoxin content in R-MLR 2006-23 was similar to that in S-MLR 2006-11 (**Table 4**). Previous work revealed that at 35°C expressions of aflatoxin biosynthesis genes, including the regulatory gene *aflR*, are not increased in comparison to expression at 30°C. Therefore, direct impact of temperature on expression of aflatoxin biosynthesis genes does not explain loss of resistance at 35°C in MLR resistant accessions (OBrian et al., 2007).

In the current study, aflatoxins were not detected in R-MLR 2006-23 at 25°C. Large concentrations of aflatoxins may be produced by *A. flavus* from 20 to 35°C and aflatoxins may be produced at temperatures as low as 11°C (Sorenson et al., 1966). The observed resistance at 25°C in the current study may be of practical importance. Resistance at moderate temperatures has not been investigated in maize germplasm identified across the globe. In addition, significantly (*P* < 0.0001) lower *A. flavus* reproduction rates occurred at 35°C in R-MLR 2006-23 compared to the other maize entries. Lower rates of epidemic increase would be supported by germplasm with reduced support for fungal reproduction (Mehl and Cotty, 2010).

Resistance with increased utility in combating mature kernel aflatoxin contamination needs to hold up under the most severe conditions favoring contamination. Mature kernel assays for resistance should include tests at elevated temperatures, like 35°C. Storage of MLR kernels for long periods may affect kernel viability and, as a result, increase risk aflatoxin contamination. Noteworthy, even at a low temperature, kernels of R-MLR 2006-23 and P33B50 were more susceptible to aflatoxin accumulation when the embryos were not viable. However, causing reduced embryo viability by inflicting distinct wounds does not always result in higher aflatoxin accumulation (**Tables 1****–****3**).

For the temperature/maize living embryo status experiment, an *A. flavus* isolate from the environment from which MLR accessions are cultivated was used. Use of endemic toxigenic strains from the target area where superior germplasm is intended to be introduced ensures assessing resistance to both fungal infection and aflatoxin contamination by local aflatoxin producers. Challenging maize germplasm against several aflatoxin-producers native to the target area would be more informative. In addition, resistance evaluations simulating daily temperature cycles at the time of crop development and/or harvest are needed. This approach has not been implemented and could provide valuable information on performance of superior resistant germplasm under realistic temperature regimes. KSA are conducted in absence of restrictions to both fungal growth and aflatoxin accumulation during at least 5 days. It would be valuable to evaluate resistance when challenged by other stresses (e.g., intra and inter species competition, insect infestations).

Results presented here demonstrate that relatively small kernel lesions significantly increase susceptibility to aflatoxin accumulation in both resistant and susceptible MLR accessions without necessarily increasing susceptibility to fungal reproduction. Similar observations have been made in other maize genotypes (Smart et al., 1990). The observed resistance was highly dependent on whole kernel integrity and presence of both wax and cuticle. Kernel damage caused by insect feeding and/or management, both pre- and post-harvest, influences aflatoxin accumulation in maize (Barry, 1997; Cotty et al., 2008; Hell et al., 2008), and our data suggests that this is true for both resistant and susceptible MLR accessions from the two examined regions of Mexico. Increased exposure to the endosperm did not increase susceptibility to aflatoxin accumulation but did favor *A. flavus* reproduction in most of the examined MLR accessions. Different tissues within the embryonic region may contribute to resistance in some MLR accessions and further research needs to be conducted to test this. In addition, presence of proteins inhibitory to *A. flavus* growth (Huang et al., 1997; Chen et al., 1998; Chen et al., 2004; Moore et al., 2007; Chen et al., 2010) and chemical characterization of wax and cutin components (Russin et al., 1997; Gembeh et al., 2001) need to be analyzed to better understand regulation of aflatoxin accumulation in MLRs. Generally, reduced sporulation on MLR accessions suggests that the accessions are less prone to infection in both field and storage conditions. Resistant MLRs from Mexico have ability to resist aflatoxin accumulation in healthy, undamaged kernels with viable embryos and/or with intact physical barriers. Whether or not resistance is maintained over prolonged contact with toxigenic fungi should be elucidated in order to facilitate selection of highly superior maize germplasm.

## Conclusion

Only a minute fraction of the great diversity of MLRs from Mexico has been examined for resistance to aflatoxins and mechanisms and quantities of resistance are expected to vary among and within MLRs. Clarifying resistance mechanisms among the vast array of MLRs may aid development of commercially useful resistant maize germplasm. Analysis of resistance dependence on temperature needs to be incorporated into final screens to determine utility of various MLR accessions. Kernel resistance under elevated temperatures will increase utility in high risk regions. There are numerous calls to integrate MLR accessions into breeding programs for maize materials with superior resistance to biotic and abiotic stresses (Mickelbart et al., 2015; Warburton et al., 2017; Mammadov et al., 2018; Mastretta-Yanes et al., 2018; Andorf et al., 2019). Results from the current study suggest maize breeding programs across the globe will benefit from exploitation of the diverse novel genetic resources that MLRs offer for aflatoxin resistance. Crucial genetic elements needed to breed stable, high yielding maize cultivars with superior resistance to aflatoxin contamination and fungal reproduction are waiting in the underutilized MLRs from Mexico.

## Data Availability Statement

The raw data supporting the conclusions of this article will be made available by the authors, without undue reservation.

## Author Contributions

AO-B and PC designed the experiments from which data are derived. AO-B conducted experiments and analyzed the data. PC provided guidance. AO-B drafted the original manuscript. All authors contributed to the article and approved the submitted version. PC and AO-B secured funds for the study.

## Funding

This research was funded by the USDA Foreign Agricultural Service, Scientific Cooperation Research Program, USDA Agricultural Research Service CRIS Project #5347-42000-02-00D, and CONACyT-Mexico Scholarship 309103.

## Conflict of Interest

The authors declare that the research was conducted in the absence of any commercial or financial relationships that could be construed as a potential conflict of interest.
